# The effect of diaries written by relatives for intensive care patients on posttraumatic stress (DRIP study): protocol for a randomized controlled trial and mixed methods study

**DOI:** 10.1186/s12912-018-0306-y

**Published:** 2018-08-16

**Authors:** Anne Højager Nielsen, Sanne Angel, Ingrid Egerod, Torben Bæk Hansen

**Affiliations:** 1 Department of Anesthesiology, Regional Hospital Holstebro, Lægårdvej 12, 7500 Holstebro, Denmark; 2 Department of Clinical Medicine, Aarhus University, Incuba/Skejby, Building 2, Palle Juul-Jensens Boulevard 82, 8200 Aarhus N, Denmark; 3 Section for Nursing, Department of Public Health, Aarhus University, Building 1260, Bartholins Allé 2, 8000 Aarhus C, Denmark; 4 Health and Medical Sciences, University of Copenhagen, Blegdamsvej 3B, 2200 Copenhagen N, Denmark; 5 Intensive Care Unit 4131, Rigshospitalet, Blegdamsvej 9, 2100 Copenhagen Ø, Denmark; 6 Regional Hospital Holstebro, University Clinic for Hand, Hip and Knee Surgery, Aarhus University, Lægårdvej 12, 7500 Holstebro, Denmark

**Keywords:** Anxiety, Depression, Intensive care, Intensive care diaries, Mixed methods, Phenomenology, Post-traumatic stress, Randomized controlled trial

## Abstract

**Background:**

Critically ill patients and their relatives have complex needs for support during their stay in the intensive care unit (ICU) and the post-ICU rehabilitation period. Diaries written by nurses have proven beneficial for patients and relatives, preventing post-traumatic stress, anxiety and depression and helping patients and families find meaning. Actively involving relatives in writing a diary for critically ill patients is a new approach to helping relatives and patients cope; however, research is limited.

The aim of this study is to test the hypothesis that a diary written by a close relative of a critically ill patient will reduce the risk of developing symptoms of post-traumatic stress disorder (PTSD) in the patient and relatives at 3 months post-ICU. Furthermore, the aim is to explore the perceptions and use of the diary and describe the diary content and structure.

**Method:**

The intervention consists of a hard-cover notebook that will be given to a close relative to write a diary for the critically ill patient while in the ICU. Guidance will be offered by ICU nurses on how to author the diary. The effect of the intervention will be tested in a two-arm, single-blind, randomized controlled trial, which aims to include 100 patient/relative pairs in each group. The primary outcome studied is symptoms of post-traumatic stress (PTSS-14). Secondary outcomes are scores on anxiety and depression (HADS) and the Medical Outcomes Study Questionnaire Short Form 36 (SF-36). The narrative structure and content of the diary as well as its use will be explored in two qualitative studies.

**Discussion:**

The results of this study will inform ICU nurses about the effects, strengths and limitations of prompting relatives to author a diary for the patient. This will allow the diary intervention to be tailored to the individual needs of patients and relatives.

**Trial registration:**

NCT02357680. Registered September 3, 2015.

## Background

### The complex rehabilitation needs of intensive care patients

ICU treatment often involves a prolonged period of ventilator dependency in a highly technical environment, resulting in a wide range of physical, cognitive and psychological impairments [1, 2]. Although many patients regain their strength within the first year, their quality of life is generally negatively affected [3–5].

Psychological problems include symptoms of post-traumatic stress disorder (PTSD), anxiety and depression. Approximately 20% of ICU survivors have clinically significant PTSD symptoms [6], consisting of re-experiencing symptoms, avoidance symptoms, arousal symptoms and cognitive symptoms [7, 8]. Ehlers and Clark [9] suggest that PTSD is characterized by the disruption of meaningful connections between events. Moreover, risk factors include having frightening or unreal experiences in the ICU [6, 10, 11], whereas accurate memories of intensive care may protect against developing PTSD [11]. The prevalence of clinically significant levels of anxiety in ICU survivors is reported to be 6.9–18% [12], and the prevalence of depression is reported to be 28% [13]. Furthermore, 50% of patients surviving critical illness do not return to school or work 1 year post-ICU [14], and impairments have a substantial effect on patients’ and their partners’ financial situations [15].

A key part of restoring quality of life after critical illness is the need to understand what has happened and why it happened. This can be difficult, as memories of intensive care may be absent, incomplete or extremely disturbed due to the critical illness and the use of sedatives. However, research show that patients continuously strive to make sense of their ICU experience [16].

### The complex needs of the vulnerable relatives of the ICU patient

When witnessing critical illness in a close family member, relatives are confronted with uncertainty about the patient’s survival [17]. During this critical period, relatives need support from healthcare professionals to reduce family stress [18]. In the rehabilitation period, the patient struggles to rebuild strength and cognitive abilities, while relatives takes the position of informal caregivers [19, 20]. Supporting the patient influences relatives’ lives, restricting daily activities and limiting the chances of keeping one’s job [21, 22]. Family members’ responses to critical illness comprise a range of psychological issues, including symptoms of PTSD (median 21%, range 13–56%) [18], depression (median 23%, range 8–42%) and anxiety (median 40%, range 21–56%) [18]. Providing a follow-up visit to the ICU could be a way of meeting family needs after discharge. Relatives have found this helpful in dealing with the ICU experience [23]. Furthermore, diaries have been described as an initiative aimed at supporting both patients and their relatives in their efforts to deal with these problems [24].

### Diaries for critically ill patients as a way to meet patients’ and relatives’ complex needs

Patient diaries written by critical care nurses have been used to help patients make sense of their ICU experience and to fill memory gaps [25, 26]. In previous research, diaries have been initiated when the length of stay was predicted to exceed 72 h and involved mechanical ventilation [27, 28]. Research has shown that nurses use their professional knowledge to create an empathic and personal story as imagined from the patients’ perspective [29].

Patients have described diaries written by nurses as helpful [29], but they particularly appreciate parts that reflect the presence of their relatives [30]. Patients and relatives have used a diary kept by nurses as a basis for dialogue about their time in the ICU [24]. This has helped relatives support patients and has promoted patient understanding of the ICU stay [24]. However, the optimal timing of the handover and reading of the diary to the patient can be difficult to determine, as the patient may feel uncomfortable if confronted with the diary too prematurely [24].

Diaries coauthored by relatives and staff have been shown to facilitate information and communication between staff and relatives [31], making ICU staff better understand relatives’ vulnerability [31, 32]. However, this has changed the objective of the diary from helping the patient to improving communication between staff and relatives. Moreover, as research has shown that relatives’ writing styles are far more emotional than nurses’ [33], relatives will be at risk of feeling exposed when co-authoring the diary with nurses. Diaries prompted by nurses but authored by relatives have enabled relatives to unload emotions, express their feelings and reflect on the critical situation [34], but patients’ perceptions of and use of such diaries have yet to be described.

### Prevention of psychological problems through the use of an intensive care diary

Diaries have been proposed as a method to prevent or reduce patients’ psychological problems, including PTSD symptoms [35, 36], anxiety and depression [37], as well as the relatives’ problems [36, 38].

The effect of diaries on ICU *patients’* psychological outcomes was studied in a randomized controlled trial (RCT) [35] wherein diaries were authored primarily by nurses, although relatives were provided with an option to participate. The diaries significantly reduced new cases of PTSD in patients (13.1% in the control group vs. 5% in the diary group, *p* = 0.02). However, this estimate may have been biased, as more cases of existing PTSD were excluded post-randomization from the intervention group (*n* = 8) than from the control group (*n* = 3). Furthermore, mean PTSD symptom scores did not differ significantly between groups [35]. A time-series-controlled study of a collaboratively authored diary showed no difference in PTSD, anxiety or depression in patients at 3 months post-ICU [36]. At 12 months post-ICU, patients in the diary group had fewer PTSD symptoms; however, the actual number of cases was not significantly reduced. Another smaller RCT [37] found that diaries authored by nurses led to a small but significant reduction in cases of anxiety and depression among patients in the diary group, whereas no significant changes were found in the control group.

The effect of diaries on *relatives* was investigated in a pilot study of patient diaries authored by nurses with optional participation for relatives [38]. This small study suggested a positive effect on relatives (median PTSS-14 scores increased from 26 to 28 in the control group and decreased from 24 to 19 in the diary group, *p* = 0.03) at 3 months post-ICU [38]. A controlled time-series study of collaboratively authored diaries [36] found a lower occurrence of PTSD (defined as the Impact of Event Scale-Revised > 22 points) among relatives using a diary than among a pre-intervention group and a post-intervention group (the diary group 31.7% vs. pre-intervention group 80% and post-intervention group 67.6%, *p* < 0.0001), but no difference at 3 months post-ICU.

### Theoretical background for the diary intervention

Diaries authored by nurses may help the critically ill patient construct an illness narrative [24]. At a more theoretical level, a narrative is described by Ricoeur as a synthesis of heterogeneous elements that transform multiple incidents into one concordant story, moving the story to its conclusion despite its discordant elements [39]. Thus, a narrative is more than a sequence of unconnected events. It is a unity in which the different elements support the conclusion of the story. Driving the story toward a preferably tidy end is not always possible [40]. Critical illness represents a disruption and fragmentation in the lives of patients and their relatives. Therefore, the illness narrative can serve the purpose of restoring a new order or identifying a new purpose instead of the tidy end, which may no longer be available or appropriate [40]. This use of narratives has been described as a way to identify or plot a livable future for severely injured patients, thus giving them hope and something for which to live [41]. Plotting a narrative can also be described as an active way of processing and understanding what is happening.

According to Ricoeur’s tri-fold mimesis [42], writing a diary may be viewed as a creative process of configuring lived experience into text [34]. However, asking the relative to author the diary may produce a narrative in the diary that is not the patient’s story. More specifically, it is the relative’s configuration of the patient’s story. However, when the relative and the patient read and discuss the diary post-ICU, this supports the patient’s configuration of his or her story. Appropriation of the text through reading opens new understandings and meanings of critical illness for both the patient and the relative [34]. This can explain how the diary relieves psychological problems common in relatives of ICU survivors.

Overall, the purpose of the ICU diary has evolved, but it has not yet been fully explored. Investigating the effect of ICU diaries authored by relatives is therefore warranted. Furthermore, a more comprehensive understanding of how a diary written by relatives is perceived by patients and relatives is needed to determine if such diaries should be implemented on a larger scale.

## Methods

### Aim

The aim of the study is to evaluate the effects of Diaries written by Relatives of Intensive care Patients in relation to the psychological recovery of both the patient and their family (DRIP study) and gain a more comprehensive understanding. The study has the following specific objectives:

- To test the hypothesis that a diary written by a close relative of a critically ill patient will reduce the patient’s risk of developing symptoms of PTSD at 3 months post-ICU;
- To test the hypothesis that a diary written by a close relative of a critically ill patient will reduce the relative’s risk of developing symptoms of PTSD at 3 months post-ICU;
- To explore how an ICU diary is perceived and used by patients and their relatives over the first year after ICU discharge;
- To explore the content and narrative structure of the diaries written by relatives for ICU patients.

### Design

The study is a single-blind, two-armed, randomized controlled trial comparing a diary intervention with standard care as control (1:1). The randomized controlled trial will be combined with two hermeneutical phenomenological studies: an interview study addressing patients’ and relatives’ perception and use of the diary and an analysis of the diary content and structure. Thus, the study is a convergent mixed methods study [43], where all studies will be conducted simultaneously, analyzed separately but where results/findings will be synthesized to arrive at a more comprehensive and complete understanding of the diary intervention [43].

### Setting

The study setting is four medical/surgical ICUs at two regional hospitals and one university hospital in Western Denmark. Levels of ICU certification among nurses range from 50 to 60% at the university hospital to more than 90% in the regional hospitals. A list of study sites can be obtained from the first author.

### Participants

#### Inclusion criteria

Patients eligible for inclusion are ≥ 18 years, expected to stay in the ICU (LOS-ICU) ≥ 48 h and expected to be mechanically ventilated ≥ 24 h. Patients with prior documented severe cognitive impairments or neurological damage will not be considered for the study. Furthermore, the patient must have one close relative willing to participate in the study. Patients must be able to read Danish, as information about the study and the questionnaire is only available in Danish.

Relatives eligible for inclusion are ≥ 18 years of age, identify themselves as a close relative to the patient (e.g., parent, spouse, partner, sibling, child or friend) and are able to read Danish. A close relative is defined here as a person having a close and personal relationship to the patient and being in contact with the patient several times during the week. Friends are eligible by this definition.

#### Exclusion criteria

Patients and relatives will be excluded from study if the patient’s LOS-ICU < 48 h or mechanically ventilated < 24 h. Relatives will not be excluded from study if the patient dies or does not consent to study participation.

### Intervention

Relatives randomized to the intervention group will receive a good quality hardcover notebook for diary use, including written information and guidance from nurses. Relatives and patients randomized to the control group will receive standard care (no diary). Written information for the relatives on how to author the diary is enclosed in the diary (see Table 1). The instructions describe the purpose of the diary as a way for the relative to help the patient after their ICU stay, as well as the potential value the diary may have for the relative. To help the relative begin writing, a list of possible topics is provided for inspiration [36] (see Table 1). The relatives are encouraged to write often and to use their own way of expressing themselves. But to avoid harming the relatives, they will be warned against writing thoughts and feelings too private or personal to be shared [44]. Finally, the leaflet will provide advice for when and how to share the diary with the patient, underscoring the need to wait until the patient agrees to see the diary to prevent the patient from being confronted with their difficult time in the ICU until he or she is ready [45].

**Table 1:** Content of the written instruction for the relative

Introduction	The aim of writing the diary is to help you (the relative) help the patient move on after intensive care. You may also find the diary beneficial. It may help you remember and process what happens.
What can you write about?	The beginning: • What happened when the patient fell ill? How did the patient get to the ICU? What did the patient do? What did you do? The time in the ICU: • What happens to the patient? What daily activities go on? Who visits the patient? • How does the patient react to treatment, information, care activities and visits? • What information do you and the patient receive? • What happens in the patient’s life, in the family and in society that the patient may find interesting? • How do you think and feel about the situation? After discharge: • How does the patient improve? • What happens in the patient’s life, in the family and in society that interests the patient?
What should you not write about?	You should not share feelings that you do not want to share with others. Remember, the diary is not a private space.
Write often	How you write the diary is your choice. You may write long or short entries as you please. The patient will surely be familiar with the way you express yourself.
Photographs	Nurses will take a minimum of two photos of the patient. These can later be placed in the diary. Photos may make a strong impression on the patient and will therefore be given directly to the patient upon consent from the patient.
Sharing the diary with the patient	When the patient is ready to receive the diary you should give the diary to the patient. Be ware that the diary can be difficult to read for the patient as the patient can have difficulties concentrating or reading. The diary will also confront the patient with a difficult time in the intensive care unit. This is why some patients may whish to delay reading the diary or reject it altogether. Do not push the patient into reading the diary before he or she is ready. Some patients like the relative to read aloud from the diary and discuss the questions it raises in the patient. When you talk about the difficult time in the ICU, it may help you both to recover from the ICU experience and move on in life.

#### Photographs

Two or more photos of each patient will be taken by the nursing staff and included in the diary [46]. One picture will be taken of the patient during the critical phase [47], showing the patient connected to the ventilator and the lines, tubing and technical equipment necessary for intensive care therapy. Additional pictures to be taken at the turning point [47], when the patient is being weaned from the ventilator or being mobilized to a chair, and possibly in the rehabilitation phase [47], will also be included. Relatives will be permitted to be photographed, staff will not. Pictures will be printed in color on semi-gloss paper (13 × 18 cm) and given to the patient by ICU nurses in a closed envelope with an explanation of what should be expected when looking at the pictures. This procedure has been chosen to avoid unnecessary discomfort, as pictures show patients in vulnerable situations. Furthermore, this procedure will allow the patient to refuse the photographs altogether, in which case photographs will be destroyed. Photographs will not be given to relatives in the case of a patient’s death.

#### Guiding relatives in writing the diary

When the relative receives the diary, an intensive care nurse will explain its use. By using clinical and communicative skills to assess the needs of the individual relative, information will be individualized, but given within the framework of the written information inserted in the diary (see Table 1). Emphasis will be on helping the relative find their own way of writing in the diary. Relatives will be encouraged to keep the diary with them and not leave it in the ICU. Furthermore, nurses will continue to guide the relative on the use of the diary when the relative visits the ICU or when nurses speak on the phone with the relative. If the conversation with the relative reveals that he or she finds it difficult to summarize the events preceding hospitalization, the nurse may suggest that the relative write about these events in keywords only or focus on the present instead. If the relative complains that they cannot find the time or space to write in the diary while in the ICU, the nurse may suggest that they write in the diary at home.

#### Training of nurses to deliver the intervention

All nurses at all sites will receive a one-hour lecture on how to deliver the intervention. This lecture will be delivered in small groups, allowing for a dialogue to evolve between the investigator (AHN) and the ICU nurses. Furthermore, ICU nurses will be offered 24-h telephone support by the investigator in case of problems relating to the delivery of the intervention.

### Outcomes

The primary measure of outcomes in the randomized controlled trial is the *Post-traumatic Stress Symptoms-14 inventory* (PTSS-14) [48]. Secondary outcomes are measured by scores on the *Hospital Anxiety and Depression Scale* (HADS) [49] and the *Medical Outcomes Study Questionnaire Short Form* (SF-36) [50]. The qualitative studies focus on the meaning that the diary holds to relatives and patients as well as describing the structure and content of the diaries [51].

### Participant timeline

#### Recruitment

**Fig. 1:**
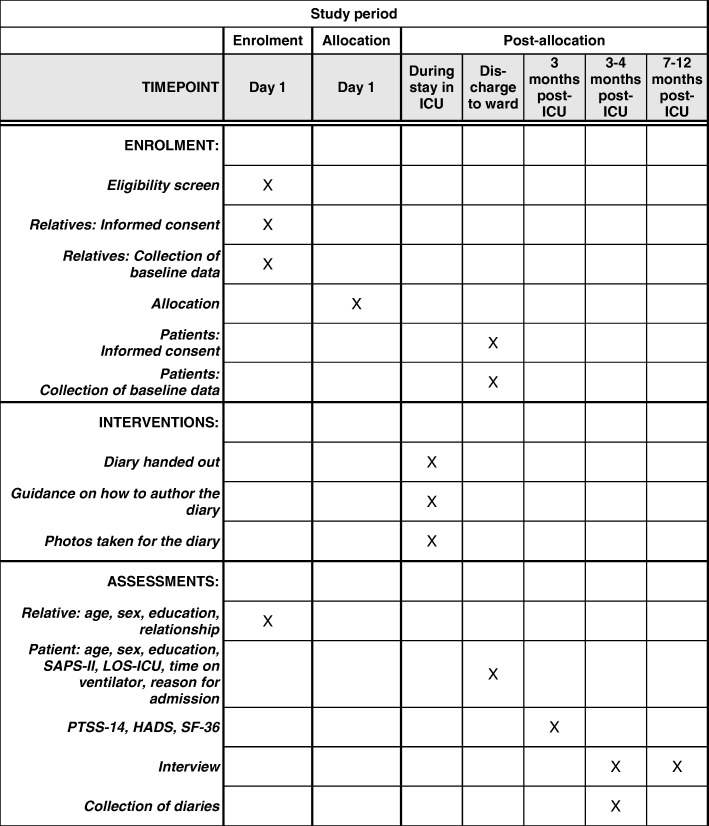
SPIRIT flow diagram

When the patient meets inclusion criteria, one close relative will be invited to participate in the study. Relatives will be randomized upon written consent and collection of baseline data to reduce the risk of selection bias. Patients will not be approached for the study until they have regained their cognitive abilities—often not occurring until after they are moved to the ward. Patient-relative dyads will be recruited for the interview study at this point, and consent to see the diary will be sought at the first interview, see Fig. 1.

#### Intervention

When allocated to the intervention group, the relative will be introduced to the diary right away, and photographing will begin. Guidance will be continued throughout the ICU stay.

Independent of the consent to participation, patients in the intervention group will be offered their photographs for the diary after discharge from the ICU. The relative has the final decision on when to share the diary with the patient.

#### Follow-up

A questionnaire will be mailed to all participants at 3 months post-ICU discharge. A reminder will be mailed 2 weeks later for non-responders to reduce attrition bias. After completion of the questionnaire, two interviews will be conducted at 3–4 months and 7–12 months post-ICU discharge with a smaller group of patients and relatives sampled for the qualitative studies. Patients and relatives will be interviewed as dyads when possible; however, relatives and patients may not be able to be interviewed together due to the late recovery of some patients or the busy schedules of participants, see Fig. 1.

### Sample size, recruitment and attrition

For the randomized controlled trial, a minimum of 71 patients in each group will be required, assuming a significance level of 5%, a power of 80%, an MCID of 4 points and a median PTSS-14 score of 24 (SD12) [35]. The Danish National Patient Registry of 2014–15 identified 3325 admissions to the study sites, out of which 1016 patients received ventilator treatment. ICU mortality was close to 30%. Based on this, we aim to include 100 patients/relatives pairs in each arm of the study. We estimate that 50% of the mechanically ventilated patients will be eligible for the study, therefore inclusion in the project is estimated to run for 30 months, starting March 2015 (see Fig. 1).

For the qualitative studies, 12 patient-relative dyads who have received the intervention will be sampled among participants in the intervention group. Patient-relative dyads will be selected to achieve phenomenological variation. This sampling strategy aims to achieve variations of experience of the phenomenon rather than variations of specific demographic variables [52]. In practice, we will approach patients and relatives consecutively as each individual has their own unique experience to share. Sampling diaries within the interview group will create strong coherent qualitative data.

### Randomization and blinding

Relatives will be randomized at a 1:1 ratio to either the intervention group or control group by the investigator or by study nurses using sealed, opaque envelopes. Block randomization ensures that each site randomizes equally to both groups. Because this is not feasible, the investigator, study nurses and participants will not be blinded to group allocation. However, to reduce the risk of detection bias, the investigator will be blinded to group allocation during data entry. The identity of each respondent will be masked and can only be re-identified using a separately kept key-file.

### Data collection and management

Patient baseline data include date of birth, age, sex, LOS-ICU, hours on ventilator, reason for admission to intensive care (medical/surgical), SAPS-II score [53] and level of education (higher, medium/low or none). Relative baseline data include age, sex, relationship to patient and level of education. Baseline data will be managed using Epidata software.

The primary outcome is measured on the PTSS-14 scale [48] developed for critically ill patients and used in studies evaluating the effect of a nurse-written diary [35, 38] on patients and relatives. The PTSS-14 is an update of the existing PTSS-10 scale based on the DSM-lll criteria, including items related to numbing and re-experiencing corresponding with the DSM-lV [48]. The PTSS-14 has been validated by Twigg et al. [48] in a cohort of patients from general ICUs in the UK. Content validity was assessed by a multidisciplinary group of experts in PTSD [48]. Criterion validity was assessed by comparing the PTSS-14 to the Post-traumatic Stress Diagnostic Scale (PDS), showing good concurrent validity with Pearson’s correlation of 0.86 at 3 months post-ICU discharge [48]. An ROC curve analysis determined 2 months post-ICU as the most favorable time to administer the PTSS-14 (AUC 0.950) [48]. However, for the diary to have time to show an effect, we will administer the PTSS-14 at 3 months (AUC 0.936, sensitivity 100%, specificity 84%) [48]. The PTSS-14 has previously been used in the RACHEL study [35, 38]. For that study [35], the PTSS-14 was translated into Danish. The process of translation involved translation back and forth, but no further validation of the Danish version. The PTSS-14 has been shown to be easy to administer to the fragile population of former ICU patients [48]. However, the fact that it is neither based on the newer DSM-V criteria nor fully validated in Danish poses some limitations. Secondary outcomes are determined by using the HADS [49] and SF-36 [50]. The HADS assesses anxiety and depression. The two scales have moderate-to-strong correlation [54], and optimal balance between sensitivity and specificity is achieved using a cut-off value of 8 for both subscales. This was done by Knowles and Tarrier [37], whereas actual cases are more conservatively estimated using a cutoff value of 11 [55]. Finally, the SF-36 will be used to measure health-related life quality. The SF-36 measures health in 8 domains [50]. Although a newer version exists (SF36 v.2), the original version will be used, as Danish reference values for this version exist [50]. Using scales to assess psychological problems like PTSD, anxiety and depression have the inherent limitation that no actual diagnosis is made; instead, we focus on reducing symptoms. Unfortunately, no general agreement exists on which outcomes to measure in trials regarding interventions to improve rehabilitation for ICU patients [55], so to ensure comparability with other studies, the PTSS-14, HADS and SF36 have been chosen. All inventories are available in Danish. They will be administered at 3 months post-ICU discharge by mail or e-mail (SurveyXact), depending on the choice of each participant. Paper questionnaires will subsequently be entered into SurveyXact by the investigator and pseudonymized.

The photocopied diaries will be transcribed verbatim, noting drawings, changing of pages, underscoring and other non-text elements. Pictures will be described in terms of content.

Interviews will be conducted at the homes of the patients and/or relatives in order to keep relatives and participants as comfortable and safe as possible. Open-ended questions will be used to explore the participants’ experiences of the diary allowing participants to recount these experiences in their own language [56]. The interviews will be digitally recorded and transcribed verbatim. Expressions of feelings will be put in parenthesis (e.g., sounds of laughter, crying, loud or muffled voices) and pauses indicated by ellipses in the transcripts [56].

### Data analysis

From the questionnaires, the total PTSS-14 mean score will be calculated for each group and compared using students’ t-test. If necessary, logarithmic transformation will be undertaken to achieve normality. The two HADS subscales describing anxiety and depression will be analyzed separately. Both subscales will be dichotomized using a cut-off value of 11. The number of cases in each group will be compared using a Chi-square test. The eight domains of the SF-36 will be calculated for each group and compared using Wilcoxon’s rank-sum test. The statistical analysis will be managed using STATA software.

In the analysis of the qualitative data (diaries and interviews) the meaning will be sought through a three-step analytic process, drawing on the interpretation theory of Ricoeur [51]. Thus, the data material will first be read thoroughly to become familiarized with it. A naïve interpretation will be formulated for each interview or diary, representing an overall naïve understanding [51] of the data. The structural analysis within the text will explain the text in terms of its smaller parts, moving from what the text says to what may be understood from the text - its reference to the world [51]. The process of explaining the text in terms of its internal relationships will serve as a validation or modification of the initially formulated naïve interpretation. This will allow the most significant interpretation to stand out in the critical interpretation. Thus, Ricoeurs’ interpretation theory will allow us to move from the specific situation of the participants to a more general and comprehensive understanding of diaries authored by relatives [51].

Subsequently, the findings of the qualitative studies and the results of the RCT will be compared. The extent to which they relate to each other will enable a more comprehensive and nuanced interpretation of the diary intervention to be made.

### Surveillance and monitoring

In order to assess the fidelity of this very complex intervention, a number of key processes will be monitored [57]. The number of eligible relative/patient pairs approached for the study will be used in monitoring recruitment to the study. After randomization, the number of pictures taken of patients in the intervention group will be documented and monitored. As part of the follow-up, the questionnaire will contain questions assessing the delivery of the intervention and its use by relatives and patients and finally, the interview study and the study of diary content and structure will also function as a sample for a qualitative evaluation of fidelity [57]. A data-monitoring committee has not been considered necessary due to the lack of competing interests and due to the small scale of the study.

### Potential harm

Should any unintended effects or adverse events be observed by nurses or reported by participants, support will be offered by the medical staff at the involved study sites. After discharge from the ICU, relatives and patients will be advised to contact the ICU or the investigator in case they experience any harm.

## Discussion

Prompting relatives to write a diary for the critically ill patient while in the ICU is a novel intervention within critical care nursing. The objective of the study is to evaluate the effects of diaries written by relatives on PTSD, anxiety and depression in both relatives and patients after the discharge from the ICU. ICU patients and relatives are in a very vulnerable situation. Before introducing a novel intervention like an ICU diary authored by relatives on a grand scale, a thorough evaluation of its effects and potential harm is necessary. We believe this study has been designed to address these questions. However, potential limitations include the lack of blinding of participants, health professionals and the investigator. This will be taken into account when interpreting the results, but it should be acknowledged that interventions of this type cannot be blinded to participants or to those delivering the intervention. The two hermeneutical phenomenological studies in combination with the larger randomized controlled trial strengthens the study as it allows us to deepen the understanding of the results achieved in the randomized trial. Furthermore, potential benefits or adverse effects may be described. Contamination between groups and lack of adherence to initial group allocation may also pose a limitation. Therefore, the degree of contamination and adherence will be assessed in the questionnaire, so this can be considered when results are interpreted.

In conclusion, this study will provide new knowledge about diaries prompted by nurses, but authored by relatives for the critically ill. Furthermore, it will allow ICU nurses to deliver effective diary interventions tailored to the individual needs of ICU patients and their relatives.

## Abbreviations

AUC: Area under Curve
DSM-III: Diagnostic and Statistical Manual of Mental Disorders, 3rd edition
DSM-IV: Diagnostic and Statistical Manual of Mental Disorders, 4th edition
DSM-V: Diagnostic and Statistical Manual of Mental Disorders, 5th edition
HADS: Hospital Anxiety and Depression Scale
ICU: Intensive Care Unit
LOS-ICU: Length of stay in the Intensive Care Unit
MCID: Minimally Clinically Important Difference
PTSD: Posttraumatic Stress Disorder
PTSS-10: Post Traumatic Stress Syndrome 10 questions inventory
PTSS-14: Post Traumatic Stress Syndrome 14 questions inventory
RCT: Randomized Controlled Trial
ROC: Receiver Operator Curve
SF-36: Medical Outcomes Study Questionnaire Short Form 36
SF-36v.2: Medical Outcomes Study Questionnaire Short Form 36 version 2
